# Efficacy of Epoetin Alfa in Managing Symptomatic Anaemia in Low-Risk Myelodysplastic Syndromes: A Retrospective Analysis

**DOI:** 10.7759/cureus.72460

**Published:** 2024-10-27

**Authors:** Mohamed Aboulela, Angela Collins

**Affiliations:** 1 Haematology, Norfolk and Norwich University Hospital, Norwich, GBR; 2 Medical Oncology, The Royal Marsden NHS Foundation Trust, London, GBR

**Keywords:** anemia, erythropoietin, haematology, malignant haematology, mds

## Abstract

Background

Myelodysplastic syndromes (MDS) are clonal myeloid disorders characterised by ineffective haematopoiesis, leading to anaemia that often requires dependence on red blood cell (RBC) transfusions. Epoetin alfa (Eprex®) is now a mainstay in the management of symptomatic anaemia in low-risk MDS patients, reducing transfusion dependence and improving the quality of life in this patient group.

Objective

This retrospective study aimed to assess the efficacy of epoetin alfa in treating symptomatic anaemia in low-risk MDS patients, focusing on transfusion independence and its relationship with baseline erythropoietin (EPO) levels and haemoglobin (Hb) response.

Methods

Data from 56 patients with low-risk MDS treated with epoetin alfa at Norfolk and Norwich University Hospital, Norwich, United Kingdom, between 2018 and 2023 were retrospectively analysed. Baseline EPO levels, transfusion history, Hb response, and the duration of transfusion independence were assessed. Statistical analyses were performed to evaluate the correlation between baseline characteristics and treatment outcomes.

Results

Among the patients, 98.2% had baseline EPO levels below the 500 IU/L threshold, with a median EPO level of 74.3 IU/L. Following an eight-week trial of 30,000 units of epoetin-alfa, 41.1% of patients showed improved Hb levels, 41.1% maintained stable Hb levels, and 17.9% experienced a decline. A significant correlation was found between lower baseline EPO levels (<250 IU/L) and a positive treatment response (p = 0.0065). Additionally, patients who required fewer transfusions before treatment had longer durations of transfusion independence (correlation coefficient = -0.40, p = 0.015). Dose escalation to 60,000 units provided a benefit to 53.3% of patients with initially stable Hb levels. The average duration of transfusion independence was 8.1 months, and patients with improved Hb levels had the longest periods of transfusion independence (p = 0.005).

Conclusion

Epoetin alfa is an effective therapy for managing symptomatic anaemia in low-risk MDS patients. This study highlights its efficacy and provides valuable predictive information, particularly showing that patients with lower baseline EPO levels are more likely to respond to treatment. While prior transfusion dependence did not significantly predict response to therapy in this cohort, it was associated with the duration of transfusion independence.

## Introduction

Myelodysplastic syndromes (MDS) are a group of clonal disorders affecting the myeloid lineage, characterised by inefficient haematopoiesis, leading to peripheral blood cytopenias and a heightened risk of progression to acute myeloid leukaemia (AML) [1]. The International Prognostic Scoring System (IPSS) categorises patients into four risk groups: low, intermediate-1, intermediate-2, and high-risk [2]. Among patients with low-risk MDS, anaemia is the predominant feature, often resulting in red blood cell (RBC) transfusion dependence, poor quality of life (QoL), and worsening of comorbidities [3].

Traditionally, erythropoiesis-stimulating agents (ESAs) such as recombinant human erythropoietin (rEPO) alfa (epoetin alfa; Eprex®) have been employed to treat anaemia in various conditions such as chronic kidney disease (CKD) and chemotherapy-induced anaemia [4]. More recently, however, epoetin alfa has demonstrated efficacy in treating symptomatic anaemia in MDS. By stimulating the proliferation of RBC precursors and inhibiting their apoptosis, epoetin alfa helps address the ineffective erythropoiesis that characterises MDS [5]. Randomised controlled trials have supported the European approval of epoetin alfa for treating symptomatic anaemia in adults with low or intermediate-1 risk primary MDS [4,6], specifically for patients with serum erythropoietin (EPO) levels below <200 IU/L and haemoglobin (Hb) levels ≤100 g/L.

Furthermore, evidence suggests that patients who respond to ESA therapy, especially those who have not required transfusions before starting treatment, have shown not only significant enhancements in QoL but also improved survival outcomes [4,7-9]. Several predictive models have been developed to assess the likelihood of response to ESA therapy. The Nordic Score considers baseline EPO levels and transfusion dependence, with lower EPO levels and fewer transfusions associated with a higher likelihood of treatment success [10].

This retrospective study aimed to evaluate the efficacy of epoetin alfa in treating symptomatic anaemia in low-risk MDS patients. Specifically, the objectives were to assess the duration of transfusion independence achieved by patients and explore any correlation between baseline EPO levels and treatment response. By analysing the treatment outcomes, the study seeks to provide insight into optimising ESA therapy in this patient population.

## Materials and methods

Study design

This retrospective study was conducted at Norfolk and Norwich University Hospital, Norwich, United Kingdom, using electronic health records (EHR) to evaluate the response of low-risk MDS patients to epoetin alfa therapy. The study focused on data from patients treated between 2018 and 2023.

Sample population

A total of 56 patients were included in the final analysis, comprising 41 male and 15 female patients, with an average age of 77 years. These patients were identified with the assistance of the hospital pharmacy, which generated reports on individuals who had been prescribed epoetin alfa within the trust.

Patients were eligible for inclusion in this study if they were diagnosed with low-risk MDS according to the IPSS and had been treated with epoetin alfa for symptomatic anaemia during the study period. Additional inclusion criteria required patients to have documented baseline EPO levels prior to the initiation of epoetin alfa therapy.

Patients were excluded from the study if they were a Jehovah's Witness, as their beliefs preclude the acceptance of blood transfusions, which could confound the analysis. Furthermore, patients who were non-compliant with epoetin alfa therapy were also excluded. Other exclusion criteria included patients who experienced acute medical admissions during the treatment period, such as those with stroke, gastrointestinal bleeding, or concurrent malignancies (e.g. palliative small cell lung cancer), which could interfere with the assessment of treatment response. Finally, patients currently undergoing epoetin alfa therapy with incomplete follow-up data were excluded from the analysis to ensure the integrity and completeness of the results.

Data collection

Data was extracted from the EHR of patients who had been prescribed epoetin alfa during the study period. In accordance with the British Society of Haematology guidelines, patients were initially started on epoetin alfa at a dose of 30,000 units weekly for an eight-week trial period. Those who exhibited a positive response to the initial dose continued on the same regimen, while non-responders were escalated to 60,000 units weekly.

Key information collected included the start date of epoetin alfa therapy, baseline EPO levels measured prior to treatment, and the number of RBC transfusions received before and after starting epoetin alfa. Hb response was categorised as follows: an improvement was defined as an increase in Hb levels of ≥5 g/L, a decline was marked by a decrease in Hb levels of more than 5 g/L, and stable Hb levels were defined as changes falling between these thresholds. Achieving transfusion independence was also considered a treatment response. Additionally, whether patients achieved transfusion independence for over three months was assessed, and the total duration of transfusion independence was recorded for responding patients.

Statistical analysis

Descriptive statistics were used to summarise the baseline characteristics of the study population. The relationship between baseline EPO levels, transfusion dependence, and treatment response was analysed using Pearson’s chi-square test and correlation coefficients. ANOVA was employed to evaluate the differences in the duration of transfusion independence between patients with differing Hb responses. Statistical significance was defined as a p-value of <0.05.

Ethics approval

This retrospective study used anonymised data extracted from EHRs, and thus formal informed consent was not required. The study was conducted in compliance with the ethical standards of Norfolk and Norwich University Hospital. Institutional Review Board approval was not deemed necessary due to the retrospective nature of the study. However, all procedures followed the institutional guidelines for data protection and patient confidentiality.

## Results

The key findings of this study are summarised in Table 1, which provides an overview of patient baseline characteristics, epoetin-alfa therapy responses, and transfusion independence outcomes.

**Table 1:** Patient baseline characteristics, epoetin alfa therapy responses, and transfusion independence outcomes EPO: erythropoietin

Date EPO started	M/F	Age in completed years when started	EPO checked pre-treatment?	If Yes, result in IU/L	Number of transfusions before EPO given	Response (improve, stable, no response)	If no positive response, dose escalated to 60,000 units weekly for eight weeks?	If no positive response after eight weeks, 60,000 units weekly stopped?	Transfusion independent >3 months?	Duration of transfusion independence (months)
January 9, 2023	M	88	Yes	66.7	2	Improvement	NA	NA	Yes	Independent
January 17, 2022	M	79	Yes	27.8	1	Improvement	NA	NA	Yes	Independent
January 7, 2019	M	85	Yes	12.4	0	Improvement	NA	NA	Yes	12
April 15, 2019	F	80	Yes	172.6	1	Stable	No	NA	No	0
January 18, 2021	M	75	Yes	91.5	2	Improvement	NA	NA	No	1
November 28, 2022	M	87	Yes	143.8	1	Improvement	NA	NA	Yes	Independent
July 3, 2020	M	65	Yes	608	2	Stable	NA	NA	Yes	12
June 24, 2019	M	80	Yes	190.3	0	Stable	Yes	NA	Yes	9
November 23, 2020	M	87	Yes	151	0	Stable	Yes	NA	Yes	13
December 23, 2019	M	64	Yes	197.2	0	Decline	Yes	Yes	No	1
February 4, 2019	M	69	No	NA	0	Stable	NA	NA	Yes	39
October 11, 2021	F	68	Yes	277	2	Decline	Yes	No	No	1
August 29, 2018	F	79	Yes	49.9	0	Improvement	NA	NA	Yes	Independent
July 2, 2018	M	84	Yes	54.3	3	Stable	Yes	Yes	No	0
May 6, 2021	F	74	Yes	80.6	0	Stable	Yes	NA	Yes	Independent
May 17, 2021	M	84	Yes	58.4	0	Improvement	NA	NA	Yes	Independent
September 30, 2019	M	74	Yes	93.1	0	Stable	Yes	Yes	No	3
May 20, 2019	M	69	Yes	177.7	2	Stable	Yes	NA	No	2
November 13, 2020	M	83	Yes	18.5	0	Improvement	NA	NA	Yes	Independent
February 14, 2022	M	69	Yes	176	2	Decline	Yes	No	No	1
March 4, 2019	M	84	Yes	65.8	0	Improvement	NA	NA	Yes	16
February 5, 2018	F	72	Yes	57.3	0	Decline	Yes	Yes	No	1
December 7, 2020	M	80	Yes	158	0	Stable	Yes	Yes	Yes	6
October 18, 2021	M	87	Yes	6	0	Improvement	NA	NA	Yes	Independent
February 12, 2018	M	77	Yes	18.2	0	Improvement	NA	NA	Yes	Independent
January 8, 2018	M	84	Yes	53.7	0	Improvement	NA	NA	Yes	30
May 24, 2021	M	78	Yes	311	2	Decline	Yes	Yes	No	0
November 21, 2022	M	72	Yes	301.4	0	Decline	Yes	Response	No	2
June 17, 2019	M	79	Yes	60.9	0	Improvement	NA	NA	Yes	15
October 3, 2022	F	76	Yes	121.8	0	Improvement	NA	NA	Yes	Independent
April 20, 2020	M	74	Yes	66.9	2	Stable	NA	NA	Yes	10
June 27, 2022	M	78	Yes	67.9	0	Stable	NA	NA	Yes	Independent
June 24, 2019	F	92	Yes	26.7	2	Improvement	NA	NA	Yes	Independent
March 9, 2020	F	78	Yes	27.4	2	Improvement	NA	NA	Yes	Independent
April 25, 2022	M	77	Yes	136	0	Stable	Yes	Stable	Yes	Independent
December 3, 2018	M	71	Yes	53.5	2	Decline	Yes	No	No	Independent
November 12, 2018	M	84	Yes	45.9	0	Improvement	NA	NA	Yes	22
April 25, 2022	M	80	Yes	328	0	Decline	Yes	Yes	No	2
December 17, 2018	F	73	Yes	14.6	0	Improvement	NA	NA	Yes	Independent
July 2, 2020	M	84	Yes	127	0	Stable	No	NA	No	2
November 16, 2020	M	82	Yes	38.7	2	Improvement	NA	NA	Yes	Independent
May 28, 2019	M	75	Yes	278.5	2	Stable	Yes	Yes	No	2
October 22, 2018	F	69	Yes	90.7	0	Improvement	Yes	Response	Yes	6
October 4, 2021	F	71	Yes	50.6	0	Stable	Yes	Response	Yes	6
January 28, 2019	M	82	Yes	104	2	Stable	Yes	No	No	0
October 28, 2019	M	74	Yes	230.2	0	Stable	Yes	Yes	Yes	3
February 5, 2018	F	86	Yes	224	2	Decline	Yes	No	No	0
September 20, 2019	M	84	Yes	66.9	0	Stable	Yes	Response	Yes	3
May 24, 2022	M	77	Yes	109	0	Improvement	NA	NA	Yes	Independent
May 16, 2022	F	63	Yes	36.9	0	Improvement	NA	NA	Yes	6
August 13, 2018	M	54	No	NA	0	Decline	Yes	No	Yes	5
July 26, 2021	M	81	Yes	101	0	Stable	Yes	Response	Yes	16
August 5, 2019	M	84	Yes	16.4	2	Stable	No	NA	No	1
January 9, 2023	M	88	Yes	92.3	0	Stable	NA	NA	Yes	Independent
October 18, 2021	F	73	Yes	36.9	0	Stable	Yes	Response	Yes	Independent
June 17, 2019	F	72	Yes	61.1	0	Improvement	NA	NA	Yes	42

Baseline EPO levels and Hb response to initial epoetin alfa therapy

**Figure 1:**
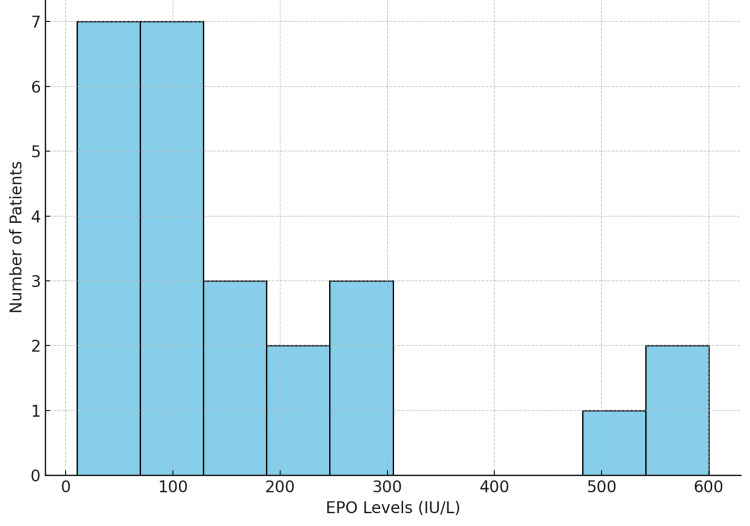
Distribution of baseline EPO levels EPO: erythropoietin

Baseline EPO levels were assessed in 96.4% of patients (n=54) before initiating epoetin alfa therapy. Among them, 98.2% (n=55) had EPO levels below the 500 IU/L threshold recommended for treatment initiation, with a median baseline EPO level of 74.3 IU/L. The distribution of baseline EPO levels is illustrated in Figure 1, showing that most patients had levels below 250 IU/L, with a few outliers above 500 IU/L.

**Figure 2:**
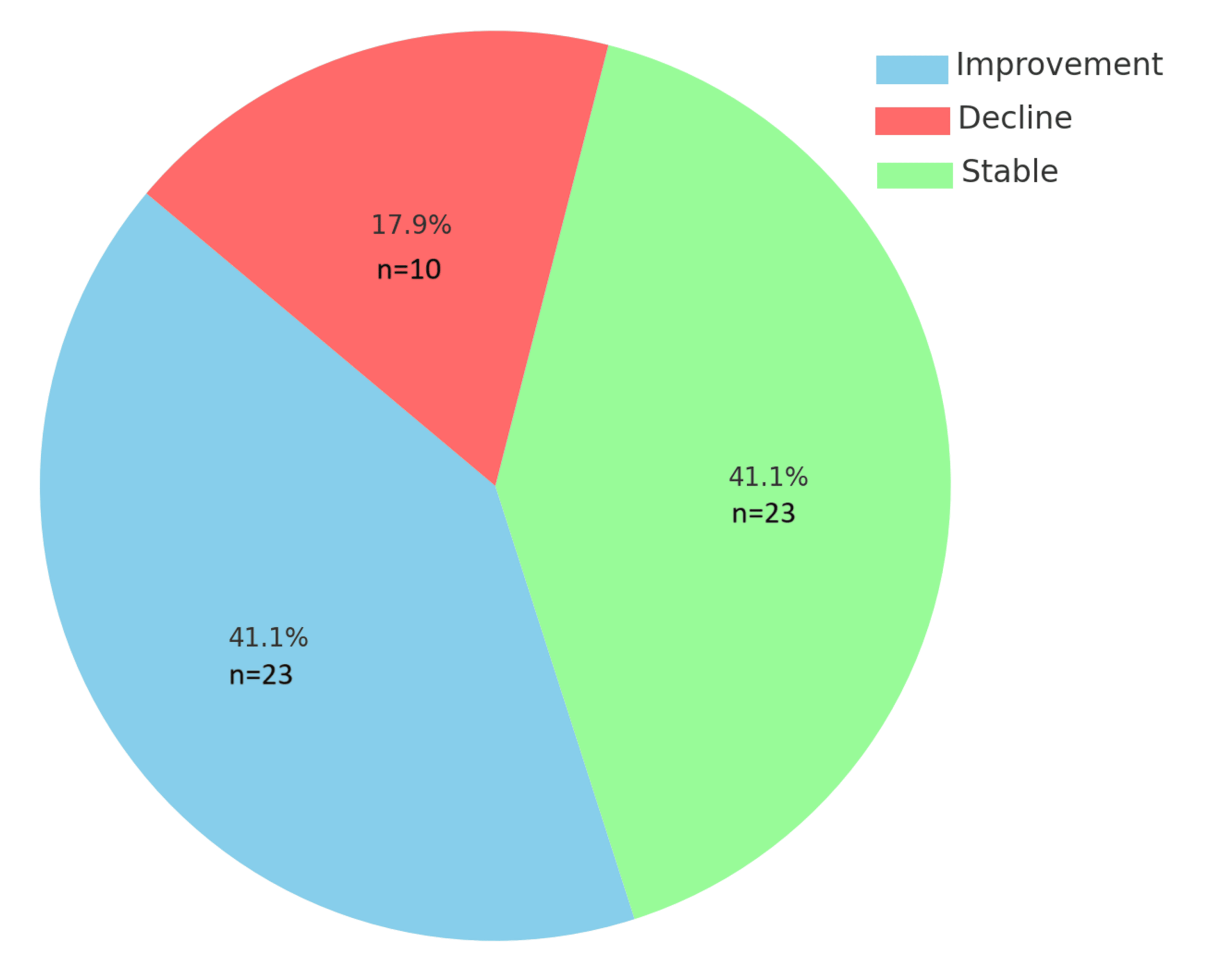
Response to initial epoetin alfa therapy

Following the initial eight-week trial of 30,000 units of epoetin alfa, 41.1% of patients (n=23) demonstrated an improvement in Hb levels, while an additional 41.1% (n=23) maintained stable Hb levels during the trial period. However, 17.9% of patients (n=10) experienced a decline in Hb levels. The overall response distribution is displayed in Figure 2.

Relationship between prior transfusions and treatment response

The number of transfusions patients received prior to starting epoetin alfa therapy was also examined. In this cohort, 55.4% of patients (n=31) required one or more transfusions before treatment, while 44.6% (n=25) did not require any transfusions.

**Figure 3:**
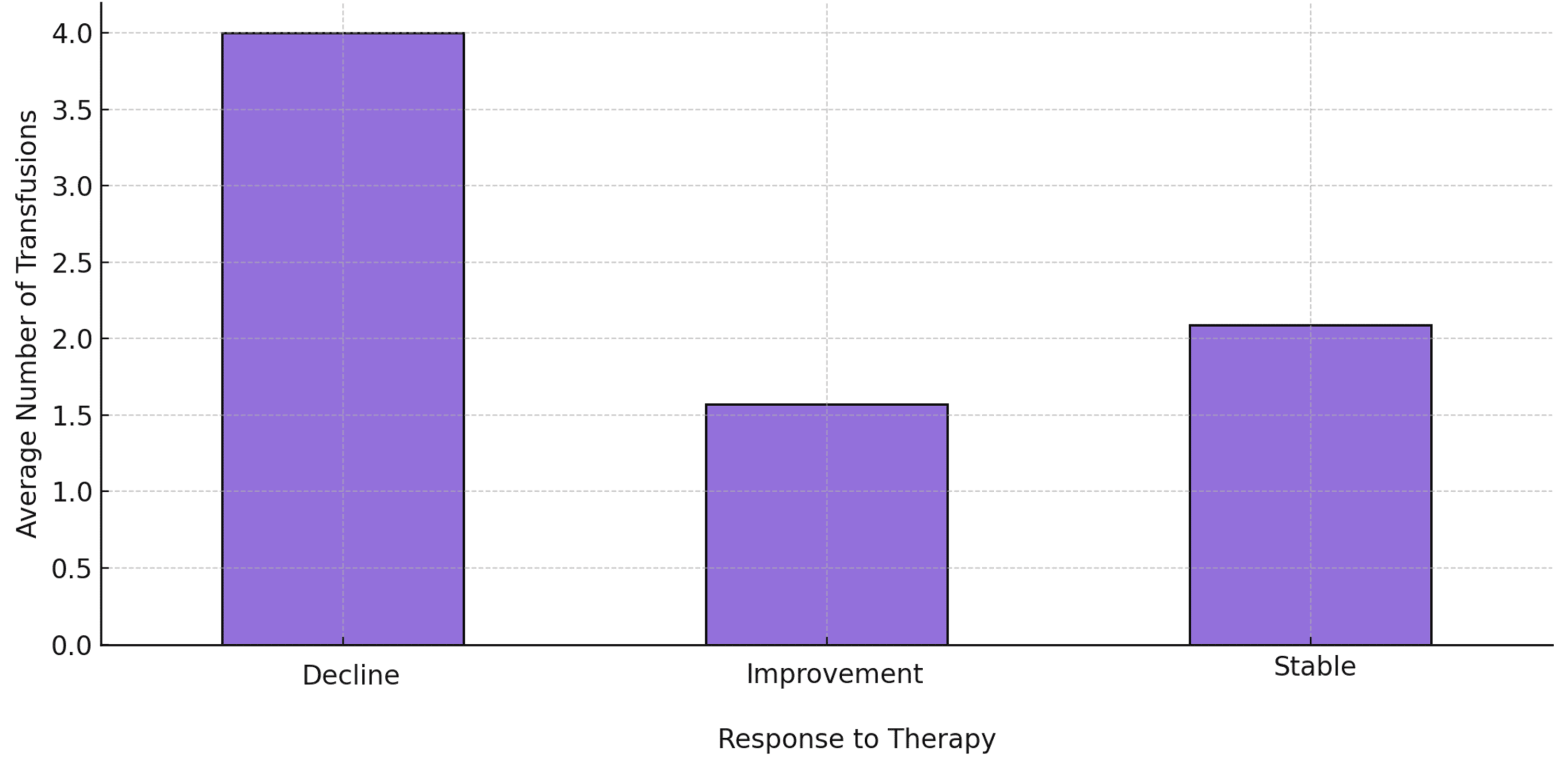
Average number of prior transfusions by treatment response

When assessing the relationship between prior transfusion status and response to epoetin alfa, no statistically significant association was found between the number of transfusions before treatment and the likelihood of response (p = 0.357). This suggests that, in this dataset, prior transfusion dependence did not have a strong influence on treatment outcomes. However, a statistically significant relationship was found between the number of transfusions received prior to epoetin alfa therapy and the duration of transfusion independence. As illustrated in Figure 3, patients who experienced a decline in Hb levels had the highest number of prior transfusions, followed by those with stable Hb levels and those with improvement.

Dose escalation to 60,000 units

Following the initial 30,000-unit trial of epoetin alfa, 25 patients were escalated to 60,000 units weekly. Among those with initially stable Hb levels, 53.3% (n=8) responded positively to the increased dose. Only one of the 10 patients with an initial decline in Hb responded after escalation. Of the patients who did not respond positively to epoetin alfa, 56% had the treatment discontinued while 44% continued therapy despite no improvement.

Transfusion independence

**Figure 4:**
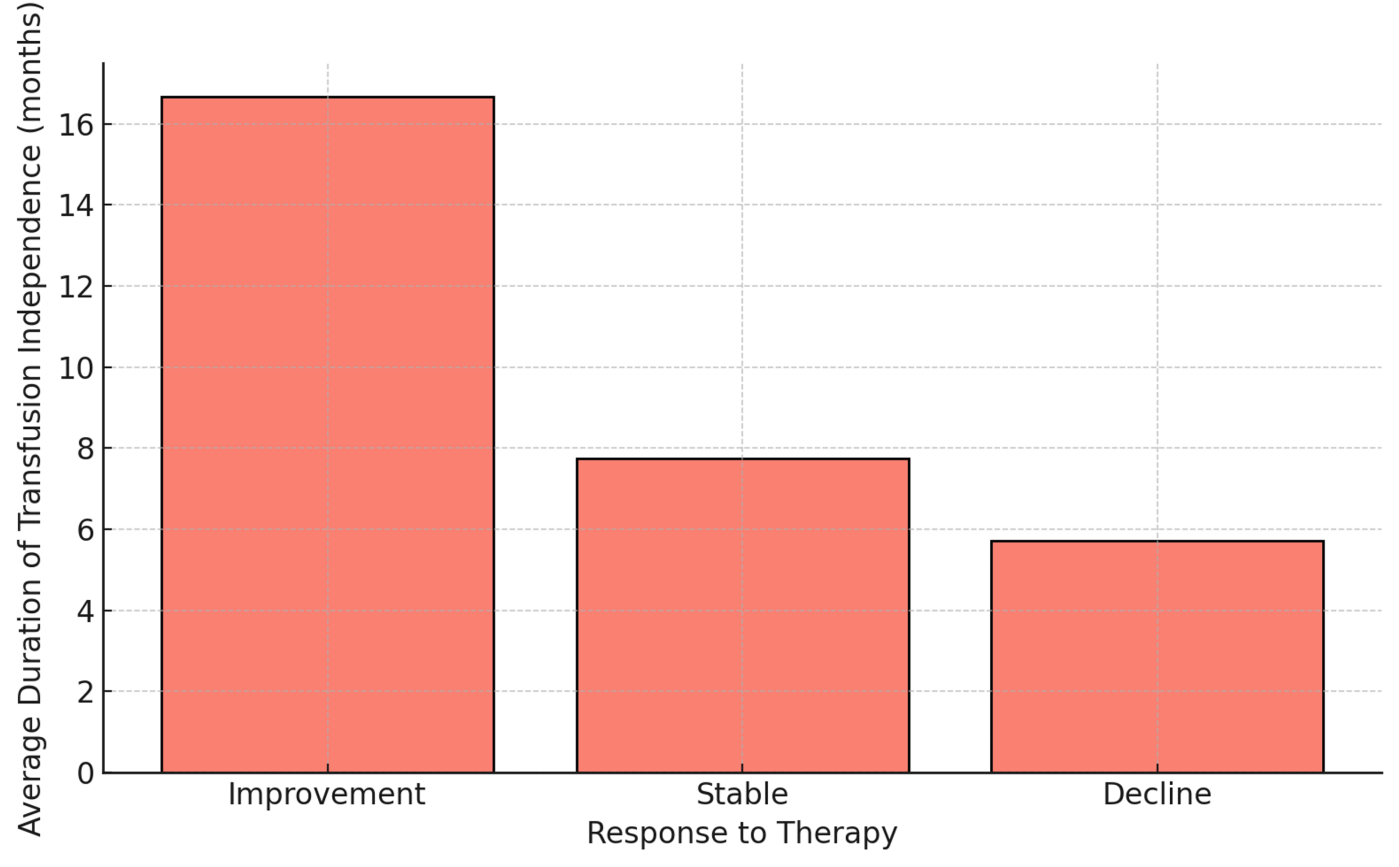
Average duration of transfusion independence by response

Transfusion independence for a duration exceeding three months was achieved in 66.1% (n=37) of the patients in this study. The average duration of transfusion independence was 8.1 months. The duration of transfusion independence also varied significantly depending on the patient's haemoglobin response to epoetin-alfa. As shown in Figure 4, patients who experienced an improvement in haemoglobin levels after epoetin alfa therapy had the longest duration of transfusion independence, followed by those with stable levels, while patients with declining Hb, as might be expected, had the shortest duration. The ANOVA test revealed a statistically significant difference in transfusion independence duration between patients who showed improvement, those who maintained stable haemoglobin levels, and those whose haemoglobin declined (p = 0.005).

Correlation between baseline EPO levels and response

Patients with baseline EPO levels below 250 IU/L had a significantly better response to epoetin alfa compared to those with levels above 250 IU/L. Of the 48 patients with EPO <250 IU/L, 43 responded to treatment, while only two of the six patients with EPO >250 IU/L responded. The chi-square test showed a statistically significant association between lower EPO levels and treatment response (p = 0.0065), indicating that lower baseline EPO levels are predictive of a better response to epoetin alfa therapy.

## Discussion

This retrospective analysis highlights the efficacy of epoetin alfa in treating anaemia in patients with low-risk MDS, particularly those with low baseline EPO levels. In our study, 41.1% of patients demonstrated a significant improvement in Hb levels with the initial 30,000-unit dose. This finding is consistent with one of the key clinical trials that led to the approval of epoetin alfa (Eprex) for the management of anaemia in low-risk MDS in Europe. In that phase 3 randomised trial, conducted by Fenaux et al., approximately 45-70% of patients treated with epoetin alfa achieved an erythroid response [4]. Additionally, a meta-analysis by Ross et al. reported overall haemoglobin response rates of 32.1% in single-arm studies and 27.3% in randomised controlled trials for epoetin-alfa [11]. This range is largely consistent with our response rates, further validating epoetin alfa as an effective treatment option for managing anaemia in low-risk MDS.

Beyond the initial response, the role of dose escalation was also examined in our cohort. Among patients who initially exhibited stable Hb levels, 53.3% responded positively to dose escalation to 60,000 units weekly. However, the benefit of dose escalation was markedly reduced in patients whose Hb levels had declined during their initial trial, with only one out of 10 patients responding. This dose escalation strategy aligns with previous studies, such as the work by Hellström-Lindberg et al., which demonstrated that higher doses of erythropoietin improved outcomes primarily in patients with stable Hb levels, though its efficacy was reduced in patients with declining levels, likely due to underlying resistance mechanisms [10].

A key finding of our study was the strong correlation between baseline EPO levels and treatment response: patients with baseline EPO levels below 250 IU/L had an 89.6% chance of responding to treatment, while those with baseline EPO levels above 250 IU/L had a 33.3% chance of responding (p = 0.0065). These results align with the findings of Park et al., who reported that patients with baseline EPO levels below 200 IU/L had a 76% response rate to erythropoietin therapy, while those with levels above 200 IU/L had a significantly lower response rate of 27% [9]. Although the threshold for EPO levels differs slightly between the two studies, the trend is consistent: lower baseline EPO levels are a strong predictor of favourable treatment outcomes in low-risk MDS patients receiving ESA therapy. This highlights the importance of assessing serum EPO levels before initiating treatment to strongly predict the likelihood of response and guide patient counselling For patients with higher baseline EPO levels, alternative treatments such as hypomethylating agents or lenalidomide may be more appropriate, as these therapies have shown efficacy in patients less likely to respond to ESAs.

In addition to baseline EPO levels, this study found that the number of transfusions received prior to epoetin alfa therapy had a significant impact on the duration of transfusion independence, with patients who required fewer transfusions before treatment demonstrating longer periods of transfusion independence following epoetin alfa therapy (p = 0.015). This finding is consistent with previous studies: Park et al. reported that patients with lower transfusion requirements prior to ESA therapy had a higher likelihood of achieving and maintaining transfusion independence for longer periods, while Fenaux et al. observed that patients without prior transfusion dependence had a 69.5% response rate to ESA therapy, compared to much lower rates in transfusion-dependent patients [4,9]. Interestingly, while prior transfusion dependence did not significantly predict overall treatment response in our study (p = 0.357), it did affect how long patients remained transfusion-independent after starting therapy. This emphasises the need for early intervention with epoetin-alfa to maximise the duration of transfusion independence, particularly in patients with lower transfusion burdens.

Transfusion independence remains a critical outcome of epoetin alfa therapy. In our study, 76.7% of responders achieved transfusion independence for an average duration of 8.1 months, which is consistent with the findings of Fenaux et al., where transfusion independence was achieved in 60-70% of responders, with a median duration of transfusion independence of approximately 11.5 months [4]. Although our cohort showed a slightly shorter duration of transfusion independence, the trend is largely comparable, indicating that epoetin alfa can provide a substantial period of relief from transfusion dependency for many patients. This relief has not only been shown in numerous studies to enhance QoL but has also been associated with reduced healthcare costs and fewer complications related to iron overload [12-14]. The importance of achieving transfusion independence cannot be overstated, as it has been consistently linked to improved survival outcomes in MDS patients, underscoring its role as a key therapeutic goal.

This study has several limitations that should be acknowledged. One of the key issues is the relatively small sample size, which may restrict the generalisability of the findings and reduce the statistical power to detect subtle differences between subgroups. Additionally, the study did not delve into the different subtypes of low-risk MDS, such as specific cytogenetic abnormalities or molecular markers, which might influence the response to epoetin-alfa therapy and offer more nuanced insights into patient stratification and outcomes. Finally, the duration of follow-up may have been insufficient to capture long-term outcomes, such as overall survival or late-onset complications, particularly in patients who initially achieved transfusion independence. Despite these limitations, this study provides valuable real-world data that help validate the findings of key clinical trials, contributing to a better understanding of the practical use of epoetin-alfa in low-risk MDS patients.

## Conclusions

Our study highlights the efficacy of epoetin alfa in treating anaemia in low-risk MDS patients, particularly in those with favourable baseline characteristics. Patients with low baseline EPO levels and no prior transfusion requirements were the most likely to benefit from therapy, exhibiting higher response rates and longer durations of transfusion independence. In contrast, patients with high baseline EPO levels and significant transfusion needs prior to therapy demonstrated poorer responses. These findings underscore the importance of using baseline EPO levels and transfusion history as key predictive markers to guide patient counselling and inform the selection of alternative therapies for those less likely to respond to epoetin alfa.
